# Nature-Based Physical Activity and Hedonic and Eudaimonic Wellbeing: The Mediating Roles of Motivational Quality and Nature Relatedness

**DOI:** 10.3389/fpsyg.2022.783840

**Published:** 2022-01-27

**Authors:** Matthew Jenkins, Craig Lee, Susan Houge Mackenzie, Elaine Anne Hargreaves, Ken Hodge, Jessica Calverley

**Affiliations:** ^1^Department of Psychological Medicine, University of Otago, Wellington, New Zealand; ^2^Department of Tourism, University of Otago, Dunedin, New Zealand; ^3^School of Physical Education, Sport and Exercise Sciences, University of Otago, Dunedin, New Zealand

**Keywords:** physical activity, motivation, nature relatedness, psychological wellbeing, eudaimonic wellbeing, hedonic wellbeing

## Abstract

The current study evaluated the degree to which nature-based physical activity (NPA) influenced two distinct types of psychological wellbeing: hedonic wellbeing and eudaimonic wellbeing. The type of motivation an individual experiences for physical activity, and the extent to which individuals have a sense of relatedness with nature, have been shown to influence the specific type of psychological wellbeing that is experienced as a result of NPA. However, the role of these two variables in the relationship between NPA and psychological wellbeing has not been examined. Thus, this study assessed the potential mediating influence of (1) motivational quality and (2) nature relatedness on the relationships between NPA and hedonic and eudaimonic wellbeing, respectively. Participants (*N* = 262) completed an online survey assessing hedonic and eudaimonic wellbeing, NPA, intrinsic motivation, autonomous extrinsic motivation, and nature relatedness. Data were analy**s**ed using Partial Least Squares Structural Equation Modelling. Results showed that motivational quality and nature relatedness both fully mediated the relationships between NPA and hedonic and eudaimonic wellbeing. Specifically, intrinsic motivation positively mediated the relationship between NPA and hedonic wellbeing. Autonomous extrinsic motivation and nature relatedness positively mediated the relationship between NPA and eudaimonic wellbeing. These findings suggest that the associations between NPA and eudaimonic wellbeing and hedonic wellbeing, respectively, are driven by different mechanisms relating to an individual’s (1) underlying motivation and (2) sense of connection to nature. These findings suggest that promoting distinct types of wellbeing (hedonic vs. eudaimonic) through NPA requires distinct approaches. Emphasising enjoyment, pleasure, and positive kinaesthetic experiences within NPA may be more conducive to hedonic wellbeing, while highlighting opportunities for connecting with nature or experiencing valued outcomes of NPA may be more conducive to eudaimonic wellbeing.

## Introduction

Spending time in natural environments has long been associated with therapeutic value, including both green spaces (e.g., forest, bush; Dadvand and Nieuwenhuijsen, 2019) and blue spaces (e.g., oceans, rivers, lakes; White et al., 2020). It has been suggested that one way by which natural environments have salutogenic health effects is by providing a platform for physical activity (van den Berg et al., 2018). Considering the well-documented health benefits of physical activity in and of itself, logically we would expect that being physically active in natural environments (nature-based physical activity; NPA) would confer additonal health benefits. As such, NPAs such as trail running, surfing, and hiking, have been researched as routes to enhanced health and in particular, enhanced mental health (e.g., Matos et al., 2017; Thomsen et al., 2018). However, while the mental health benefits of NPAs are well-documented (Thompson Coon et al., 2011; Lawton et al., 2017), understanding of the mechanisms underlying the relationship between NPA and mental health is still in its infancy.

Mental health can be been described as lying on a continuum, ranging from mental illness (e.g., depression, anxiety) to high levels of psychological wellbeing (Keyes, 2002). While NPA has been discussed as a way of managing, treating, and preventing mental illness (Maier and Jette, 2016), researchers have also called for a focus on its capacity to enhance psychological wellbeing (Seligman and Csikszentmihalyi, 2000; Jeste et al., 2015; Zhang and Chen, 2019) and to examine the mechanisms through which physical activity creates its mental health effects (Lubans et al., 2016). While psychological wellbeing is characterised by both *hedonic* and *eudaimonic* wellbeing (Diener, 1984), research has not established their respective relationships with NPA, nor the potential socio-psychological pathways that may explain these relationships.

Hedonic wellbeing (HWB) relates to the subjective experiences of pleasure and enjoyment. Hedonic wellbeing is characterised by high levels of positive affect and low levels of discomfort, and is often equated with happiness (Diener, 1984; Kahneman, 1999; McDowell, 2010). On the other hand, eudaimonic wellbeing (EWB) – coming from the word “daimon”, or “true self” – is characterised by experiences of meaning, purpose, flourishing, self-discovery, and reaching one’s potential (Waterman, 1993; Deci and Ryan, 2008; Huta and Ryan, 2010; Waterman et al., 2010).

While HWB and EWB are highly interrelated and likely influence one another (e.g., Fredrickson, 2004), they are associated with different motivation for physical activity and physical activity experiences (Pritchard et al., 2020). EWB stems from activities that an individual finds personally meaningful or promote growth, which can often be challenging and not inherently pleasurable (Waterman, 1993). Thus, an individual may actively choose to forgo pleasurable experiences (i.e., HWB) in order to experience EWB. This proposition is supported by evidence suggesting that increases in EWB can be accompanied by reduced HWB (Ryan and Deci, 2001; McMahan and Estes, 2011). Although there is limited research with regards to the respective benefits of HWB and EWB, these two types of wellbeing have been suggested to have distinct long-term health consequences. For example, EWB has been associated with reduced psychological distress and morbidity, and has been shown to be a buffer to disadvantages that are usually associated with poorer physical health, such as lower education levels (Henderson et al., 2013; Ryff, 2017). In contrast, Henderson et al. (2013) showed that HWB is associated with increased positive affect, vitality, and life satisfaction, and that hedonic-oriented (i.e., pleasure-seeking) behaviours are associated with reductions in negative affect, depression, and stress. EWB stemming from meaningful experiences has been shown to have longer lasting outcomes as compared to HWB coming from hedonic or pleasurable experiences (Huta and Ryan, 2010). Thus, although both HWB and EWB are associated with overall psychological wellbeing and mental health benefits, they each offer distinct benefits. Experiencing and integrating both types of wellbeing contribute to what psychologists have termed “flourishing” to describe a state of positive mental health (Henderson and Knight, 2012), which itself is associated with less impairment, less societal burden, decreased disability, decreased rates of physical illness and healthcare utilisation, and increased psychosocial functioning (Keyes and Annas, 2009).

Being physically active in natural environments such as green or blue spaces has been associated with increases in both HWB and EWB. For example, walking in green spaces been shown to result in enhanced mood and vitality, key markers of HWB, as compared to walking in urban environments (e.g., Hartig et al., 2003). Passmore and Howell (2014) also demonstrated that a 2-week NPA intervention resulted in increased EWB and HWB, and in a series of survey studies, Wolsko et al. (2019) found that NPA was associated with both EWB and HWB. However, other studies have found no significant difference between NPA and non-NPA with regards to EWB (e.g., Lawton et al., 2017) or HWB (e.g., Jenkins et al., 2021), respectively. While these mixed findings could be a result of different populations being sampled or different measurement tools (as suggested by Pritchard et al., 2020), it is also possible that intervening socio-psychological factors may influence the relationships between NPA and HWB and EWB, respectively. Two potential variables that may mediate these relationships during NPA are: (1) *motivational quality*, specifically the extent to which motivation for physical activity aligns with HWB or EWB outcomes; and (2) the extent to which an individual experiences a *connection to nature*.

The concept of motivational quality reflects the notion that there may be a variety of reasons underlying a given behaviour, which are located along a continuum of self-determination (Deci and Ryan, 2002). Within the framework of Self-Determination Theory, motivational quality is described as being either *controlled* or *autonomous* (Ryan and Deci, 2017). Controlled motivation originates either from externalised sources resulting in behaviours undertaken out of obligation due to external rewards or punishments, or internalised sources where behaviours are undertaken due to self-imposed pressure (e.g., according to societal norms concerning body image). In contrast, autonomous motivation is characterised by a sense of volition, choice, and self-endorsement when engaging in an activity (Deci and Ryan, 2002) and has been associated with positive psychological wellbeing on the basis of being aligned with one’s authentic self (Ryan and Deci, 2017). Autonomous motivation can be further divided into *intrinsic* or *extrinsic* forms. Intrinsic motivation refers to undertaking an activity for its own sake out of enjoyment or pleasure, thus making the activity inherently rewarding. Autonomous extrinsic motivation refers to behaviours that are undertaken because they align with an individual’s sense of self which (known as integrated regulation) or because of the valued outcomes that are achieved (known as identified regulation) (Ryan and Deci, 2017). An example of integrated regulation is an individual regularly running because they identify themselves as being a “runner,” while someone experiencing identified regulation may be active because they value being healthy or it gives them an opportunity to spend valued time with family.

While it is suggested that *all* types of autonomous motivation are associated with psychological wellbeing in general (Ryan and Deci, 2001), we expected to find specific relationships between intrinsic motivation and HWB, and autonomous extrinsic motivation and EWB, due to their respective conceptual alignments. For example, the defining characteristics of intrinsic motivation - feelings of pleasure and enjoyment - directly align with HWB. Meanwhile, undertaking an activity because it has outcomes that are personally meaningful (i.e., autonomous extrinsic motivation) is aligned with EWB. Thus, in the context of NPA, we might expect that if an individual’s NPA drives their autonomous extrinsic motivation, this may result in EWB. On the other hand if, they are intrinsically motivated, then HWB might be an expected outcome.

While intrinsic motivation and autonomous extrinsic motivation often co-occur in an individual’s motivational profile, this is not always the case (Lindwall et al., 2017). Living a life that is aligned with one’s values is not always synonymous with undertaking pleasure and enjoyment-oriented behaviours. Indeed, undertaking behaviours that we value can sometimes be a source of discomfort and have the potential to lead to reduced HWB (Hayes et al., 2011). This notion of motivational quality resulting in distinctive psychological outcomes in the context of NPA has been signalled in previous research. For example, in a cross-sectional study, Jenkins et al. (2021) showed that intrinsic motivation mediated the relationship between NPA and HWB, but autonomous extrinsic motivation did not (EWB was not measured in Jenkins et al.’s study). Thus the question remains as to whether autonomous extrinsic motivation contributes to the EWB outcomes of NPA.

The extent to which we connect or relate to our natural environments may also influence psychological wellbeing outcomes. Nature relatedness is defined as “the subjective sense of connection people have with the natural environment” (Nisbet and Zelenski, 2014, p.184). Nature relatedness is often used interchangeably with the term “nature connectedness,” which is defined as “an individual’s subjective sense of their relationship with the natural world” (Pritchard et al., 2020, p.1145). As these two terms are conceptually analogous, we use the term *nature relatedness* in the current study.

Our sense of relatedness with nature and how this influences our psychological wellbeing has been described in various ways, including through biophilic (Ulrich, 1993) and self-identification (Schultz and Tabanico, 2007) perspectives. Biophilic accounts highlight that for much of our evolutionary history, we have spent considerable time within nature and thus have a strong affiliation, connection, or love (“philia”) for nature (Ulrich, 1993). Consequently, spending time in nature is, or at least can be, inherently enjoyable and is associated with considerable health benefits (Seymour, 2016). In the self-identification approach, an individual may see nature as a strong part of their identity and are therefore driven to spend more time in nature (Schultz and Tabanico, 2007). Interestingly, these two approaches – the biophilia and self-identity – also align with the intrinsic motivation and autonomous extrinsic motivation through love of an activity (in this case, spending time in nature), and valuing and identifying as a person who spends time in nature, respectively. Logically we would therefore expect that there are wellbeing benefits which arise from this sense of nature relatedness and motives for spending time in nature due to either an underlying affiliation with natural environments or because we self-identify with nature (Grinde and Patil, 2009; Ryan et al., 2014).

Simply spending time in nature has been shown to increase nature relatedness (Nisbet et al., 2011). The extent to which NPA results in increased levels of nature relatedness may influence the psychological outcomes of NPA (Wolsko and Lindberg, 2013). In a recent meta-analysis, Pritchard et al. (2020) showed that nature relatedness had significant effects on both HWB and EWB. Across a series of five correlational studies, Wolsko et al. (2019) also found that time spent in nature – especially in NPA - and nature relatedness were associated with EWB. Pritchard et al. argued that nature relatedness was an important predictor of EWB due to associations with the types of personal growth-related experiences that promote EWB. Mayer and Frantz (2004) further suggested that NPA encourages a sense of “community, kinship, embeddedness, and belongingness” (p.512), which Wolsko et al. (2019) pointed out are also central characteristics of eudaimonia.

Although Pritchard et al.’s (2020) meta-analysis showed no significant difference in terms of relationship strength between EWB and HWB, respectively, and nature relatedness, these authors acknowledged that variations in EWB measurement across studies may have masked potential differences in their results. With respect to HWB, Howell et al. (2011) found that HWB (“feeling good”) was less reliably associated with nature relatedness than EWB (“functioning well”). Considering the evidence for positive associations between nature relatedness and HWB and EWB, respectively (albeit mixed in the case of HWB), and that spending time in nature increases nature relatedness, it is possible that nature relatedness is an important mediator of the relationship between NPA and psychological wellbeing. If nature relatedness does indeed mediate this relationship, this means that we should promote nature relatedness as an important aspect of NPA in order to obtain psychological wellbeing benefits, by purposely incorporating ways in which individuals can connect to nature while being active. Therefore, further research examining the relationships among NPA, nature relatedness, and HWB and EWB, respectively is needed.

Research investigating the relationships amongst nature relatedness, specific types of motivational quality, and psychological wellbeing is scarce, yet investigating these relationships may provide a more nuanced understanding how NPA fosters hedonic and eudaimonic psychological wellbeing. Identifying the mechanisms that explain how NPA inflences HWB and EWB, respectively (i.e., *via* nature relatedness, autonomous extrinsic motivation, or intrinsic motivation) may illuminate multiple avenues for attaining overall psychological wellbeing. Research on these relationships has implications for both our theoretical conceptualisations and practical approaches to promoting NPA and psychological wellbeing.

### Research Questions

The current study sought to address the following research questions:

(1) To what extent does the amount of NPA undertaken predict HWB and EWB, respectively?
(2a) To what extent do autonomous extrinsic motivation and intrinsic motivation separately mediate the relationship between NPA and HWB?
(b) To what extent do autonomous extrinsic motivation and intrinsic motivation separately mediate the relationship between NPA and EWB?
(3a) To what extent does nature relatedness mediate the relationship between NPA and HWB?
(b) To what extent does nature relatedness mediate the relationship between NPA and EWB?
(4) To what extent do motivational quality and nature relatedness collectively influence HWB and EWB, respectively?

### Hypotheses

H1a. NPA will significantly predict HWB.

H1b. NPA will significantly predict EWB.

H2a. Intrinsic motivation will partially mediate the relationship between NPA and HWB.

H2b. Autonomous extrinsic motivation will *not* mediate the relationship between NPA and HWB.

H3a. Autonomous extrinsic motivation will partially mediate the relationship between NPA and EWB.

H3b. Intrinsic motivation will *not* mediate the relationship between NPA and EWB.

H4a. Nature relatedness will partially mediate the relationship between NPA and HWB.

H4b. Nature relatedness will partially mediate the relationship between NPA and EWB.

## Materials and Methods

### Research Design and Participants

This study utilised a cross-sectional survey design, hosted by the online survey platform Qualtrics. Participants were identified from a pool of respondents to a previous cross-sectional survey study, which investigated the determinants and outcomes of physical activity as a result of the COVID-19 pandemic (Jenkins et al., 2021). This previous study used a convenience sampling method to recruit a sample of participants across the New Zealand population *via* mainstream social media platforms (Akers and Gordon, 2018), email lists and newsletters for universities and other large organisations (e.g., regional and national sports organisations, city councils), and a virtual snowball recruitment technique (Baltar and Brunet, 2012). At the end of this original study, participants were asked if they were willing to participate in a follow-up study, and those who responded “yes” were invited to complete the current survey. Thus, the inclusion criteria reflected those of the original study: participants were required to be aged 18 years or older, and were excluded if they had any contraindications (e.g., illness, injury) that prevented physical activity. These criteria were assessed *via* screening questions at the beginning of the survey.

The survey was open for 9 days between April 20th and April 28th, 2021. The survey took an average of 12 min to complete. Ethical approval was obtained prior to data collection from a university ethics committee. All participants provided online informed consent before completing the survey.

### Measures

#### Eudaimonic Wellbeing

The Questionnaire for Eudaimonic Wellbeing (QWEB; Waterman et al., 2010) consists of 21 items, each answered on a 5-point Likert scale from 0 (strongly disagree) to 4 (strongly agree). Total score range is 0 to 84. Example items include: “My life is centred around a set of core beliefs that give meaning to my life,” “It is more important that I really enjoy what I do than that other people are impressed by it,” and “I believe it is important to know how what I’m doing fits with purposes worth pursuing.” Seven of the 21 items are negatively worded, and were reverse scored. Previous research has shown that the QWEB demonstrates good reliability and validity scores (Cronbach’s Alpha = 0.86; Waterman et al., 2010). Although originally devised and validated as a one-factor scale by Waterman et al. (2010), subsequent research has shown that a four-factor solution also fits, comprising of factors pertaining to sense of purpose, engagement in rewarding activities, living from beliefs, and effortful engagement (Schutte et al., 2013). For the purpose of the current study, the four-factor solution was used.

#### Hedonic Wellbeing

The World Health Organisation Wellbeing Index (WHO-5; World Health Organisation, 1998) is a self-report measure of hedonic wellbeing, containing five items scored on a Likert-type scale ranging from 0 (at no time) to 5 (all of the time). The item stem “Over the last 2 weeks” is followed by items such as: “I woke up feeling fresh and rested” and “I have felt calm and relaxed.” The WHO-5 has been shown to have high reliability and validity across many different samples in different countries (Topp et al., 2015).

#### Physical Activity Motivation

The Behavioural Regulation in Exercise (BREQ-3-PA version; Markland and Tobin, 2004) consists of 24 items, each answered on a 5-point Likert-type scale from 0 (not true for me) to 4 (very true for me). For the purposes of this study, we only used data from the intrinsic motivation, identified regulation and integrated regulation subscales of the BREQ-3-PA. A two-factor structure was used consisting of intrinsic motivation (four items; e.g., “I am physically active because I enjoy it”) and autonomous extrinsic motivation (eight items from the identified and integrated regulation subscales; e.g., “It’s important to me to be regularly physically active,” “I consider physical activity as part of my identity”). This two-factor structure has been shown by previous research to have high reliability and validity (MacDonald’s ω = 0.93) (Jenkins et al., 2020).

#### Nature Relatedness

The Nature Relatedness scale (NR scale; Nisbet et al., 2009) consists of 21 items, each scored on a 5-point Likert scale from 1 (disagree strongly) to 5 (“agree strongly”), eight of which are reverse-scored. There are three dimensions to the scale: self-identity (eight items; e.g., “My connection to nature and the environment is a part of my spirituality”), experience (six items; e.g., “I enjoy being outdoors, even in unpleasant weather”), and perspective (seven items; the self in the wider environmental context), and results are reported using the overall NR score. When assessing the NR scale for reliability and validity *via* hierarchical component model analysis (Hair et al., 2017), items on the “perspective” subscale failed to meet acceptable cut-off validity criteria. For this reason, and because this factor was not conceptually related to our aims of examining nature relatedness through nature based physical activity, this factor was omitted from our analyses.

#### Weekly Nature-Based Physical Activity

Nature-based physical activity was measured using a combination of two measures. First, participants completed the International Physical Activity Questionnaire-Short Form (IPAQ-SF; Craig et al., 2003). The IPAQ is a 7-item measure of self-reported physical activity, measuring the amount of moderate- and vigorous-intensity physical activity, walking, and sitting undertaken by participants over the previous 7 days. Example items include: “During the last 7 days, on how many days did you do vigorous activities like heavy lifting, exercise classes, or fast cycling for at least 10 min at a time?” followed by the question: “How much time did you usually spend doing vigorous physical activities on one of those days?” In accordance with IPAQ scoring guidelines (Craig et al., 2003), Metabolic Equivalent Unit (MET) minutes were calculated from participants’ responses by multiplying the number of minutes reported undertaking each intensity by 3.3 (for light walking), by 4 (for moderate intensity PA), and 8 (for vigorous physical activity). When summed, this results in the number of weekly MET minutes undertaken by an individual. The IPAQ-SF has demonstrated good validity and consistency (e.g., Lee et al., 2011).

Second, participants were asked: “What proportion (percentage) of the physical activity you have undertaken over the past 7 days was performed in the following locations?” followed by the options: “In an indoor setting outside of the home (e.g., gym, swimming pool, and sports hall),” “Indoors at home (e.g., home gym),” “Outdoors in a non-nature setting (e.g., streets, cycles lanes, sports fields),” and “In a nature-based setting (e.g., forest, ocean, and beaches).” Participants were asked to assign a percentage to each of these locations (between 0 and 100%). This measure has been used previously to measure NPA (Pasanen et al., 2014). Weekly NPA was then determined by multiplying the percentage of time spent undertaking physical activity “in a nature-based setting” by the weekly total MET minutes calculated within the IPAQ-SF.

### Data Cleaning

For the IPAQ-SF, data screening, cleaning, and coding were undertaken according to Craig et al.’s (2003) detailed guidelines. This included the truncation of data points indicating more than 960 min of physical activity per week as these are suggested to be outliers (Craig et al., 2003). Very low missing data were present for the items used in the structural model (<5% missing data for each variable), and the Little’s Missing Completely at Random test produced a non-significant result, meaning data was missing completely at random. Thus, any missing data was estimated using the Expectation-Maximisation algorithm (Peters and Enders, 2002).

### Data Analysis

**FIGURE 1:**
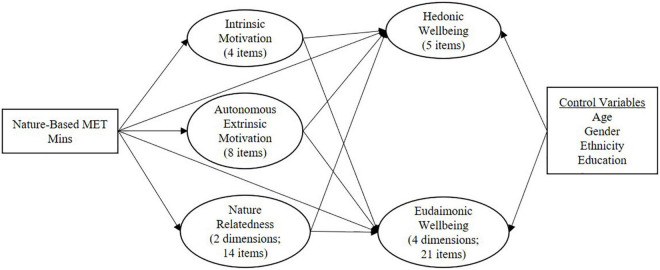
Tested relationships amongst variables.

Partial Least Squares Structural Equation Modelling (PLS-SEM) using SmartPLS (v. 3.2.7) (Ringle et al., 2018) was used for data analysis. PLS-SEM was used over covariance-based SEM (CB-SEM) due to the main objective of this study being the prediction of hedonic and eudaimonic wellbeing instead of theory testing and model validation (which is usually the focus of CB-SEM) (Hair et al., 2017). Also, PLS-SEM allows issues with regards to sample and sub-sample size limitations to be overcome when dealing with complex models. Thus, PLS-SEM was used as the model to be estimated was complex relative to sample size, comprising twelve latent constructs (inclusive of hierarchical component models and control variables) with multiple indicators (Haenlein and Kaplan, 2004). The model tested for various relationships amongst nature-based MET minutes, intrinsic motivation, autonomous extrinsic motivation, nature relatedness, hedonic wellbeing, and eudaimonic wellbeing. This model can be seen in Figure 1. Variables controlled for in the analysis included gender, ethnicity, age, and education. For the purposes of the analysis, education was estimated as an ordinal variable and binary categories were used for gender (male/female) and ethnicitiy (NZ European/Non-NZ European), as PLS-SEM cannot be estimated for categorical variables with more than two categories. Binary categories were considered the most appropriate way to include these variables in the analysis because of their distribution in Table 1. As 99.2% of the data were represented by the male and female category, the authors deemed the binary categorisation appropriate for this analysis. As 82.9% of the sample was categorised as NZ European, and the distribution of other ethnicities was relatively even and small, the authors deemed that having a binary variable representing NZ European and non-NZ European would be appropriate for this analysis. The four control variables were then included in the model as predictors of the final dependent variables (i.e., HWB and EWB) to account for their potential influences on the parameter estimates of the variables of interest.

**TABLE 1:** Descriptive statistics.

Variable	Category	N (%)
Gender	Male	70 (27.8)
	Female	180 (71.4)
	Gender diverse	1 (0.4)
	Prefer not to say	1 (0.4)
Education	No formal qualification	1 (0.4)
	Less than high school	1 (0.4)
	High school graduate	13 (5.2)
	Some tertiary education	14 (5.6)
	Certificate or diploma	26 (10.3)
	University undergraduate degree	64 (24.3)
	Postgraduate degree or above	133 (52.8)
Ethnicity	NZ European	209 (82.9)
	Mâori	2 (0.8)
	Samoan	1 (0.4)
	Cook Island Mâori	1 (0.4)
	Indian	1 (0.4)
	Other	38 (15.1)
Age	Mean = 44.80 (S.D. = 13.8), Lowest age = 20 years, Highest age = 73 years	
NPA (MET mins)	Mean = 574.17 (S.D. = 809.59) Skew = 1.991 Kurtosis = 4.4	
Percentage of time undertaking NPA	Mean = 27.4%; S.D. = 30.8%	

### Sample Size Adequacy

*A priori* and *post-hoc* power analyses using G*Power was used to determine the adequacy of the sample size (Faul et al., 2009). Using a suggested minimum *R*^2^ value of 0.10, a statistical power of 95%, and eight predictors (the HWB construct has the largest number of predictors) (Cohen, 1988), the *a priori* G* Power calculation indicated that a minimum sample size of 213 would be required. In addition, the *post-hoc* G* Power calculation for a minimum *R*^2^ of 0.10, a sample size of 262 (the number of usable responses obtained), and eight predictors indicated that the statistical power achieved using the study’s sample size was 0.98, which is sufficiently above Cohen’s (1988) recommendations, thus justifying the adequacy of our sample size.

## Results

### Demographic Characteristics

The obtained sample was *N* = 262. Participants’ mean age was 44.8 years (range = 20–73 years, SD = 13.8). They were predominantly female (71.4%), and NZ European (82.9%; 0.8% identified as Mâori; 15.1% identified as Other). Over half the sample had completed some form of postgraduate education (52.8%). Descriptive statistics, including mean scores for NPA and a correlation matrix among latent constructs, can be seen in Table 1 and Appendix Table A1.

### Outer Model Evaluation (Measurement Models)

#### First Order Latent Constructs

Internal reliability of the unidimensional reflective constructs (intrinsic motivation, autonomous extrinsic motivation, HWB) were supported, all with Cronbach’s α and Composite Reliability scores above 0.7 (Hair et al., 2017) (see Table 2). Indicator reliability was supported, with all item loadings above 0.7. Convergent validity was supported, with all AVE values above 0.5. Finally, discriminant validity was supported as all constructs’ HTMT confidence intervals did not include 1.

**TABLE 2:** Measurement model evaluation.

Latent construct	Indicator	Mean	S.D.	Skew	Kurtosis	Outer loading	Cronbach’s α	Composite reliability	AVE	HTMT	*R* ^2^	*Q* ^2^
Intrinsic motivation (IM)	IM 1	3.223	0.955	−1.393	1.9	0.917	0.936	0.954	0.838	Does not include 1	0.085	0.069
	IM 2	3.023	0.986	−0.946	0.648	0.935						
	IM 3	2.855	1.099	−0.872	0.226	0.908						
	IM 4	3.137	0.975	−1.174	1.191	0.902						
Autonomous extrinsic motivation (AEM)	AEM 1	3.019	1.21	−1.183	0.452	0.882	0.933	0.945	0.682	Does not include 1	0.097	0.064
	AEM 2	3.579	0.716	−2.014	4.928	0.768						
	AEM 4	2.669	1.261	−0.652	−0.568	0.766						
	AEM 4	3.594	0.697	−1.975	4.805	0.734						
	AEM 5	3.011	1.095	−1.027	0.303	0.857						
	AEM 6	3.479	0.819	−1.636	2.44	0.856						
	AEM 7	2.565	1.417	−0.614	−0.972	0.865						
	AEM 8	2.604	1.376	−0.677	−0.802	0.863						
Nature relatedness (NR)	NR 1	n/a	n/a	−0.679	0.098	0.918	0.801	0.909	0.834	Does not include 1	0.128	0.106
	NR 2	n/a	n/a	−0.965	0.997	0.908						
Hedonic wellbeing (HWB)	HWB 1	2.264	1.377	−0.033	−0.996	0.747	0.830	0.879	0.591	Does not include 1	0.305	0.159
	HWB 2	3.414	1.133	−0.706	0.007	0.731						
	HWB 3	3.498	0.929	−1.206	1.655	0.803						
	HWB 4	3.172	1.056	−0.742	−0.038	0.741						
	HWB 5	2.751	1.224	−0.511	−0.39	0.820						
Eudaimonic wellbeing (EWB)	EWB 1	n/a	n/a	−0.454	−0.161	0.851	0.802	0.872	0.631	Does not include 1	0.341	0.201
	EWB 2	n/a	n/a	−0.422	0.270	0.794						
	EWB 3	n/a	n/a	−0.311	−0.223	0.833						
	EWB 4	n/a	n/a	−0.755	1.246	0.689						

#### Higher Order Latent Constructs

The higher order latent constructs of nature relatedness (two dimensions) and EWB (four dimensions) were assessed for reliability and validity using the disjoint two-stage approach (Sarstedt et al., 2019). First, the lower-order components of nature relatedness and EWB were included in the model and directly linked to all other constructs that the higher-order constructs were theoretically linked to (i.e., NPA to the two dimensions of nature relatedness, the nature relatedness dimensions to HWB and the four dimensions of EWB, all IVs to four dimensions of EWB). Next, the construct scores of the lower-order components (i.e., the two dimensions of nature relatedness and four dimensions of EWB) were saved and then used as indicators of the higher-order constructs (i.e., two indicators for nature relatedness, four indicators for EWB). In this way, the reliability and validity of higher-order reflective-reflective type constructs can be assessed using the usual approach to assessing reflective constructs in PLS-SEM (Sarstedt et al., 2019). Internal reliability of the higher order constructs was supported, all with Cronbach’s α and Composite Reliability scores above 0.7 (Hair et al., 2017) (see Table 2). Indicator reliability was supported, with all item loadings at or above 0.7. Convergent validity was supported, with all AVE values above 0.5. Finally, discriminant validity was supported as all constructs’ HTMT confidence intervals did not include 1.

### Inner Model Analysis: Direct, Indirect, and Mediation Analysis

No collinearity issues between predictor constructs were detected as all VIF values were below 5 (Hair et al., 2017). Thus, assessment of the path estimates could continue. Concerning control variables, only age had significant and positive associations with HWB and EWB. Review studies have shown that wellbeing is closely linked to age, and evidence suggests that wellbeing improves with advancing age (Steptoe et al., 2015). This underscores the importance for controlling this variable in the analysis.

Table 3 shows the path estimates from the analysis of the direct and indirect effects. NPA was not a significant direct predictor of HWB or EWB, leading us to reject H1a and H1b. The mediation analysis showed that intrinsic motivation fully mediated the relationship between NPA and HWB. The results also showed that autonomous extrinsic motivation and nature relatedness fully mediated the relationship between NPA and EWB. In all of cases of mediation, we expected partial, not full mediation. Therefore H2, H3, and H4 were partially supported.

**TABLE 3:** Path estimates amongst variables.

Path	Path coefficient	Effect size (*f*^2^)	95% CI
NPA → Intrinsic motivation	**0.291**	**0.093**	**0.209–0.376**
NPA → Autonomous extrinsic motivation	**0.311**	**0.107**	**0.231–0.383**
NPA → Nature relatedness	**0.357**	**0.146**	**0.264–0.442**
NPA → Hedonic wellbeing	0.043	0.002	−0.063–0.149
NPA → Eudaimonic wellbeing	−0.050	0.003	−0.150–0.053
Intrinsic motivation → Hedonic wellbeing	**0.319**	**0.050**	**0.133–0.506**
Intrinsic motivation → Eudaimonic wellbeing	0.126	0.008	−0.068–0.327
Autonomous extrinsic motivation → Hedonic wellbeing	0.117	0.007	−0.075–0.302
Autonomous extrinsic motivation → Eudaimonic wellbeing	**0.271**	**0.039**	**0.077–0.468**
Nature relatedness → Hedonic wellbeing	0.081	0.007	−0.048–0.209
Nature relatedness → Eudaimonic wellbeing	**0.216**	**0.049**	**0.082–0.344**
NPA → Intrinsic motivation → Hedonic wellbeing	**0.093**	**n/a**	**0.038–0.159**
NPA → Intrinsic motivation → Eudaimonic wellbeing	0.037	n/a	−0.020–0.102
NPA → Autonomous extrinsic motivation → Hedonic wellbeing	0.037	n/a	−0.022–0.097
NPA → Autonomous extrinsic motivation → Eudaimonic wellbeing	**0.084**	**n/a**	**0.024–0.151**
NPA → Nature relatedness → Hedonic wellbeing	0.029	n/a	−0.017–0.077
NPA → Nature relatedness → Eudaimonic wellbeing	**0.077**	**n/a**	**0.029–0.129**
**Control variables**			
Age → Hedonic wellbeing	0.212	0.060	0.112–0.313
Age → Eudaimonic wellbeing	0.207	0.060	0.099–0.306
Gender → Hedonic wellbeing	−0.040	0.002	−0.139–0.062
Gender → Eudaimonic wellbeing	0.003	0	−0.106–0.114
Ethnicity → Hedonic wellbeing	−0.006	0	−0.132–0.113
Ethnicity → Eudaimonic wellbeing	0.050	0.003	−0.065–0.16
Education → Hedonic wellbeing	0.021	0.001	−0.090–0.131
Education → Eudaimonic wellbeing	0.083	0.010	−0.029–0.19

*Significant relationships are shown in bold.*

**FIGURE 2:**
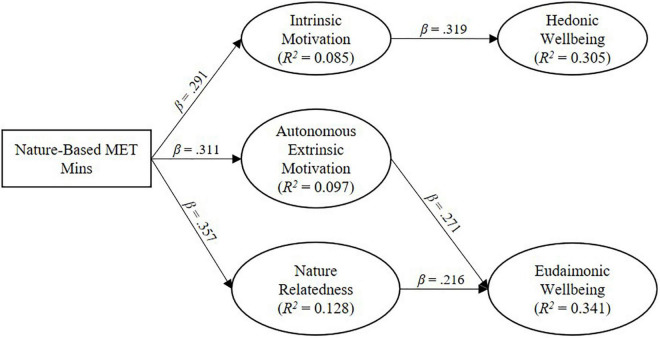
Figure showing only significant positive associations.

Figure 2 summarises the significant associations found in the study. Together, autonomous extrinsic motivation and nature relatedness accounted for 34.21% of the variance in EWB. Intrinsic motivation accounted for 30.5% of the variance in HWB. NPA accounted for 8.5% of the variance in intrinsic motivation, 9.7% of the variance in autonomous extrinsic motivation, and 12.8% of the variance in nature relatedness.

## Discussion

The purpose of this study was to to investigate the relationships between NPA and hedonic and eudaimonic wellbeing, in addition to assessing the potential mediating influences of motivational quality and nature relatedness. Contrary to our hypotheses, NPA did not directly predict HWB nor EWB. Instead, NPA predicted HWB and EWB through particular types of motivation (intrinsic motivation and autonomous extrinsic motivation) and nature relatedness. For each of the mediating variables identified in this study we hypothesised partial mediation. However, the results showed full mediation in all of these cases. Therefore, our results suggested that NPA can affect psychological wellbeing only *through* specific motivational types and/or nature relatedness, but that NPA does not directly affect psychological wellbeing. These findings extend the literature on NPA and psychological wellbeing, which has thus far shown mixed results. While some studies have reported direct positive relationships amongst NPA and psychological wellbeing, others have not found direct relationships amongst these variables (Pritchard et al., 2020). Thus, our study elucidates on the intervening variables that facilitate the influence of NPA on psychological wellbeing.

The relationship between NPA and HWB was fully mediated by intrinsic motivation, while the relationship between NPA and EWB was fully mediated by autonomous extrinsic motivation and nature relatedness. Although further research is required to discern *why* the relationships between NPA and EWB and HWB were mediated through different forms of motivation, it is possible that an individual’s reasons for participating in NPA facilitate different attentional foci during the activity. Specifically, if you have intrinsic motivation for NPA, then you may be primarily focused on the immediate experiences of enjoyment and pleasurable affective responses during these activities, which are characteristic of hedonic wellbeing. There is already convergent support from previous findings for this conjecture. NPA has been consistently associated with enjoyment and intrinsic motivation (e.g., Thompson Coon et al., 2011; Lahart et al., 2019). Furthermore, biophilic theories of nature engagement (e.g., Ulrich, 1993) posit that humans have a strong connection for nature and thus spending time in nature is inherently pleasurable. Waterman et al. (2008) also showed that intrinsic motivation was more consistently associated with HWB than with EWB.

In contrast, if you have autonomous extrinsic motivation for NPA, then you may be primarily focused on achieving important goals or valued outcomes of NPA, and/or a sense of meaning/connection derived from being active in nature (also inherent in nature relatedness, all of which are conceptually congruent with eudaimonic wellbeing (e.g., Huta and Ryan, 2010). Furthermore, identity theorists (e.g., Schultz and Tabanico, 2007) propose that natural environments offer opportunities for reflection, which may in turn increase the salience of one’s core values or identity (Kaplan, 1995). Thus, individuals who identify as someone affiliated with nature may undertake NPA in order to affirm their identity (i.e., autonomous extrinsic motivation), which is linked to EWB. Thus although there is less empirical evidence to support our NPA-autonomous extrinsic motivation/nature relatedness-EWB proposal, the conceptual link is evident. Vallerand (2000) has argued that experiences of different forms of motivation at the situational level (during the activity) feed into motivation held at the contextual level (for NPA in general). Therefore, one way to examine these conjectures further would be to measure motivation at the situational rather than contextual level (as we did here) to see if these relationships are stronger.

Our results also expanded on previous findings that simply spending time in nature increases one’s sense of nature relatedness (Nisbet et al., 2009), by suggesting that this is also the case when people are *moving* through nature, and that increases in nature relatedness are associated with increases in EWB. However, our prediction that nature relatednesss would positively mediate the relationship between NPA and HWB was not supported and indeed, nature relatedness did not directly predict HWB. Although Pritchard et al.’s (2020) meta-analysis showed that nature relatedness has been associated with both EWB and HWB, other researchers have found that nature relatedness was more reliably associated with EWB than with HWB (e.g., Howell et al., 2011). This distinction in relationships may be influenced by several factors, including the sense of connection, belonging or identity discussed above (Wolsko et al., 2019), which often characterises eudaimonia.

The different mediating pathways found in the results supported our hypotheses in that the distinct motivational qualities (intrinsic motivation, autonomous extrinsic motivation) associated with NPA were associated with different types of psychological wellbeing (HWB and EWB, respectively). Overall, these findings aligned with previous studies which concluded that being in nature is associated with internally driven (autonomous) motivation, but not with ego-driven (controlled) motivation (Calogiuri and Elliott, 2017). The findings also build on previous research showing that intrinsic motivation for physical activity, but not autonomous extrinsic motivation, had positive associations with HWB (Jenkins et al., 2021). However, Jenkins et al.’s (2021) study did not include measures of EWB. Therefore, the current results expand our understanding of these relationships and offer further insights into the relationships between different types of psychological wellbeing and different types of motivation, specifically in the context of NPA. Certainly, the role of intrinsic and autonomous extrinsic motivation, both in relation to NPA and as mediators on the pathway from NPA to psychological wellbeing, warrants further investigation, preferably with measures that have been developed specifically to assess motivation for nature-based activities.

Although more research is needed to ascertain causal relationships, these findings suggest that there are distinct motivational pathways through which NPA influences different facets of psychological wellbeing. Indeed, finding full mediation implies that we have uncovered potential mechanisms that may explain how NPA influences psychological wellbeing (both EWB and HWB). While the results indicate a lack of direct relationship between NPA and psychological wellbeing, motivational quality and nature relatedness are intervening variables that help to explain how NPA can lead to improvements in psychological wellbeing. This is particularly pertinent if a specific type of psychological wellbeing is sought. For instance, it has been suggested that EWB may be longer-lasting than HWB (Huta and Ryan, 2010). Thus, promoting the valued outcomes of NPA or demonstrating how these activities support an individual’s identity, has the potential to foster EWB. Additionally, encouraging the development of a connection to nature (i.e., fostering nature relatedness) may also promote the attainment of EWB. For example, emphasising individuals’ values regarding health-promoting behaviours (e.g., physical activity) and pro-environmental behaviours, and connecting these values *via* nature-based physical activities (e.g., beach clean-ups, tree planting, “plogging”) might be practical ways of supporting both autonomous extrinsic motivation and nature relatedness. These suggestions should be treated with caution as further research is required to understand causality amongst these variables. However, future investigations in this vein are warranted as they have the potential to engender increases in physical, psychological, and planetary health (Kruize et al., 2019). The multiple benefits of these approaches suggest that integrating these types of interventions could potentially enhance the value of current mainstream public and environmental health initiatives.

In addition to the implications for promoting EWB, the current study has implications for promoting HWB in NPA contexts. While EWB may be longer lasting than HWB, the importance of HWB for overall psychological wellbeing is still significant (Disabato et al., 2016). Thus, for individuals who are are more motivated by opportunities for enjoyment than by health-related values, emphasising the pleasurable aspects of NPA (Williams and Bohlen, 2019) such as positive emotions or kinaesthetic and other sensory stimulation, has the potential to foster HWB. On an individudal level, this might mean tailoring NPA activities to match an individual’s activity preferences in order to maximise enjoyment. From a health promotion perspective, affording access to immediately enjoyable (i.e., hedonic) NPA experiences might be one important route to enhancing psychological wellbeing across broader populations.

### Strengths and Limitations

These results expand our understanding of the relationships amongst motivational quality, nature relatedness, and NPA. In addition, they support the propositions of existing theory – namely the association between motivational quality and psychological wellbeing (Deci and Ryan, 2008). With regard to limitations, the majority of participants were female, educated to postgraduate level, and highly active. These characteristics limit the extent to which the study findings can be generalised to populations with different demographics, particularly with regard to physical activity levels. In addition, it could be argued that participants were highly motivated to complete the survey, as participants had completed a previous study and then agreed to further contribute to the current research project. While this is not a limitation of the study design itself, it may limit the conclusions draw from the data and indicates that these reults require further investigation across diverse populations. Finally, the measure of motivation used was for physical activity overall, not specifically *NPA*. Thus, modifying measures such as the BREQ-3 to reflect NPA might be a consideration for future research in this area. Finally, the exclusion of items pertaining to the perspective factor (i.e., wider connections to nature) were omitted primarily on the basis of not meeting validity criterion in the measurement model. While this was a valid approach, we recognise that it limits our conclusions with regards to an individual’s wider environmental connections to nature. Further research that incorporates measures of perspective (while being psychometrically valid) would address this limitation.

### Future Research

Future research should investigate the direction of causality underlying the relationships identified in this study. This might be achieved by developing targeted interventions specifically designed to promote intrinsic motivation, autonomous extrinsic motivation, and nature relatedness, and then assessing whether changes in these variables leads to distinct psychological wellbeing outcomes. This could involve, for instance, the development and/or evaluation of integrative NPA programmes focused on environmental conservation activities which emphasise nature connection and exploring one’s own values in relation to nature-based experiences in order to increase EWB. Considering current global environmental challenges (Intergovernmental Panel on Climate Change [IPCC], 2021), promoting such programmes has the potential to serve not only public health goals (i.e., mental health and physical health), but also to support increased guardianship of the environment. Developing and evaluating NPA programmes that emphasise hedonic NPA experiences, such as enjoyment or kinaesthetic sensory sensations (Allen-Collinson and Leledaki, 2015), might be explored in their capacity to increase HWB. Further research could also investigate if the findings apply to enhancing HWB and EWB in a broader range of populations beyond those included in the current study (e.g., youth, or those living with compromised mental health).

Researchers might also consider exploring more nuanced definitions of NPA in future studies, including distinct environments (blue spaces vs. green spaces) and the level of nature immersion (e.g., viewing from a distance vs. immersing onself in the natural environment). For example, previous studies have suggested that blue spaces might be more strongly associated with psychological wellbeing than green spaces (e.g., White et al., 2010; Nutsford et al., 2016). Future investigations should explore how these distinctions amongst different environments or experience characteristics influence the relationships found in the current study. Similarly, accounting for frequency, intensity, time, and type (FITT) principles of physical activity might highlight how these specific factors influence psychological wellbeing outcomes in nature-based settings.

## Conclusion

It is important to note that physical activity does not need to be undertaken in nature for it to be beneficial to psychological wellbeing. Indeed, gaining access to natural environments may be unfeasible and/or unattractive for some individuals or populations (Rigolon et al., 2021). Yet the potential benefits, both for individuals and environments, that NPA may offer appear sufficient to consider increased promotion of NPA where possible, if such promotion also supports autonomous motivation and/or nature relatedness. This research found that both EWB and HWB were influenced by the type of motivation and the extent of nature relatedness underpinning NPA participation. The results of this study provide a basis to formulate further hypotheses regarding the nuanced relationships between NPA and psychological wellbeing, and to promote NPA as a way of attaining both physical and mental health benefits.

## Data Availability Statement

The datasets presented in this study can be found in online repositories. The names of the repository/repositories and accession number(s) can be found below: http://doi.org/10.6084/m9.figshare.17126912.

## Ethics Statement

The studies involving human participants were reviewed and approved by the Human Ethics Committee, University of Otago. The patients/participants provided their written informed consent to participate in this study.

## Author Contributions

MJ, SH, EH, KH, and JC conceived the study. MJ, JC, and EH coordinated the data collection. CL, MJ, and JC cleaned and analysed the data. MJ led the manuscript writing and all authors contributed to the final submission.

## Conflict of Interest

The authors declare that the research was conducted in the absence of any commercial or financial relationships that could be construed as a potential conflict of interest.

## Publisher’s Note

All claims expressed in this article are solely those of the authors and do not necessarily represent those of their affiliated organizations, or those of the publisher, the editors and the reviewers. Any product that may be evaluated in this article, or claim that may be made by its manufacturer, is not guaranteed or endorsed by the publisher.

## Appendix

**TABLE 4:** Correlation matrix among latent constructs.

	Autonomous extrinsic motivation	Eudaimonic wellbeing	Hedonic wellbeing	Intrinsic motivation	Nature relatedness	Nature-based MET mins
Autonomous extrinsic motivation	1					
Eudaimonic wellbeing	0.473	1				
Hedonic wellbeing	0.446	0.487	1			
Intrinsic motivation	0.800	0.448	0.484	1		
Nature relatedness	0.401	0.423	0.328	0.445	1	
Nature-based MET mins	0.311	0.181	0.228	0.291	0.357	1
